# Spatial mapping of long-term recrudescent herpes simplex labialis

**DOI:** 10.1016/j.jdcr.2021.09.040

**Published:** 2021-10-12

**Authors:** David Paslin, Jules Perret, Carlton Pennypacker

**Affiliations:** a Department of Dermatology, University of California, San Francisco, San Francisco, California; b Paris-Saclay University, Gif-sur-Yvette, France; c Lawrence Berkeley National Laboratory, and University of California, Berkeley, Berkeley, California

**Keywords:** aging, herpes, HSV-1, labialis, long term, mapping, natural history, neurons, recrudescent, recurrent, simplex, spatial, trichloroacetic acid, virus, HSV, herpes simplex virus, HSV-1, herpes simplex virus type 1, IL, interleukin, TCA, trichloroacetic acid

## Introduction

Herpes simplex virus (HSV) type 1 (HSV-1) infections, both primary and reactivation, have been repeatedly studied, but only for short periods of time.^1^ The long-term (years) natural history of recrudescent (clinically apparent) outbreaks of oral-labial HSV infection is not well characterized.^1^ We have found no report that describes the long-term; (ie, >6 months), continuous natural history of labial HSV-1 clinical expression. This case study helps to fill that knowledge gap in that it records the frequency of HSV-1 outbreaks over a span of 38 years and precise location of outbreaks over a span of 11 years.

## Case report

A 79-year-old immunocompetent subject (a dermatologist) observed the onset of clinically apparent labial HSV at the age of 19. His primary infection had been subclinical. Subsequently, HSV-1 disease was confirmed serologically. He recorded the frequency of recrudescent eruptions of HSV-1, from 42 to 79 years of age (1983-2020), and drew rough maps of sites of the outbreaks. With millimeter precision, he drew detailed maps of the HSV-1 outbreaks, allowing exact localization of each outbreak over a period of 11 years (2008-2018). Exhaustive searches of the literature show that no other such long-term monitoring and careful measurements of HSV-1 outbreaks has ever occurred. In addition, the spatial recurrence of the events may shed some light on how the recrudescences can re-emerge in the same physical site for years.

During the first 11 years of overt disease, the subject had applied trichloroacetic acid (TCA) 100% (wt/vol) to many of the affected sites, but he observed neither increased nor decreased frequency of outbreaks from its use. Size, duration, and location of outbreaks remained unchanged. During the subsequent 27 years of observation, the subject avoided any therapeutic intervention enabling a 38-year, long-term recording of the natural history of recrudescent labial HSV, premised on the lack of impact of the initial TCA use. Outbreaks occasionally followed sun exposure or upper respiratory viral infections but mostly followed fatigue and lack of sleep.

The subject observed 177 recurrences during those 38 years with a mean of 4.66 episodes per year. t test comparison of the frequency of outbreaks during the first 19 years with the second 19 years resulted in a probability (*P*) value of .058. When comparing frequency of outbreaks in quartiles by analysis of variance, in the first quartile (9.5 years), there were 52 episodes with a mean of 5.47 episodes per year; in the second quartile 55 episodes, with a mean of 5.79; in the third quartile 42 episodes, with a mean of 4.42; and in the fourth quartile 28 episodes, with a mean of 2.95 (*P* = .01).

**Fig 1:**
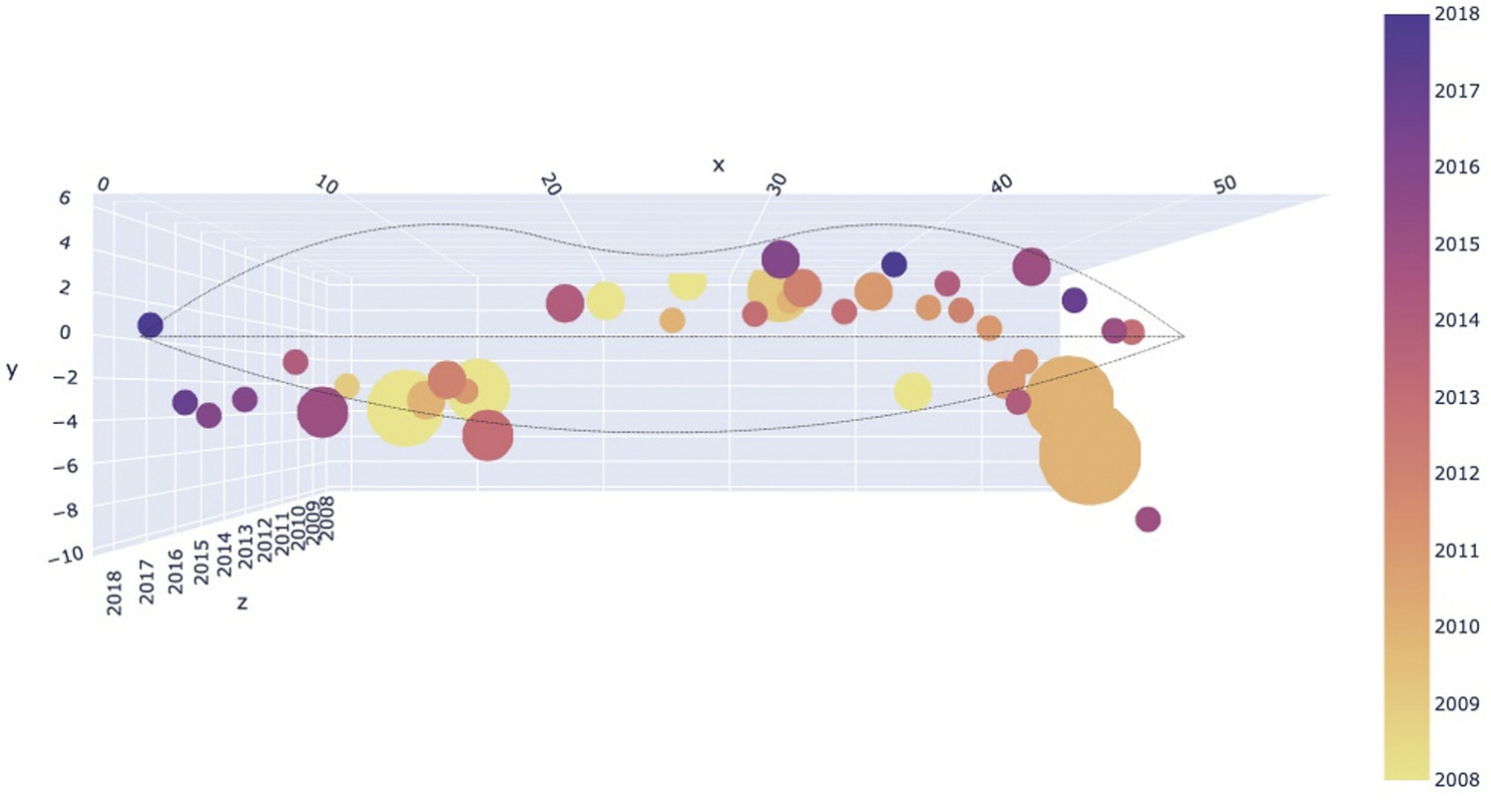
Herpes simplex virus type 1. Projection of view of outbreaks. The size of the bubbles is proportional to the size of the lesion. A few bubbles cover and obscure others. This explains why 44 recurrences were identified, whereas Fig 1 shows only 41 bubbles. The time axis is projected into the page, and x and y coordinates are in the plane of the page. More active sites with multiple events are apparent here.

**Fig 2:**
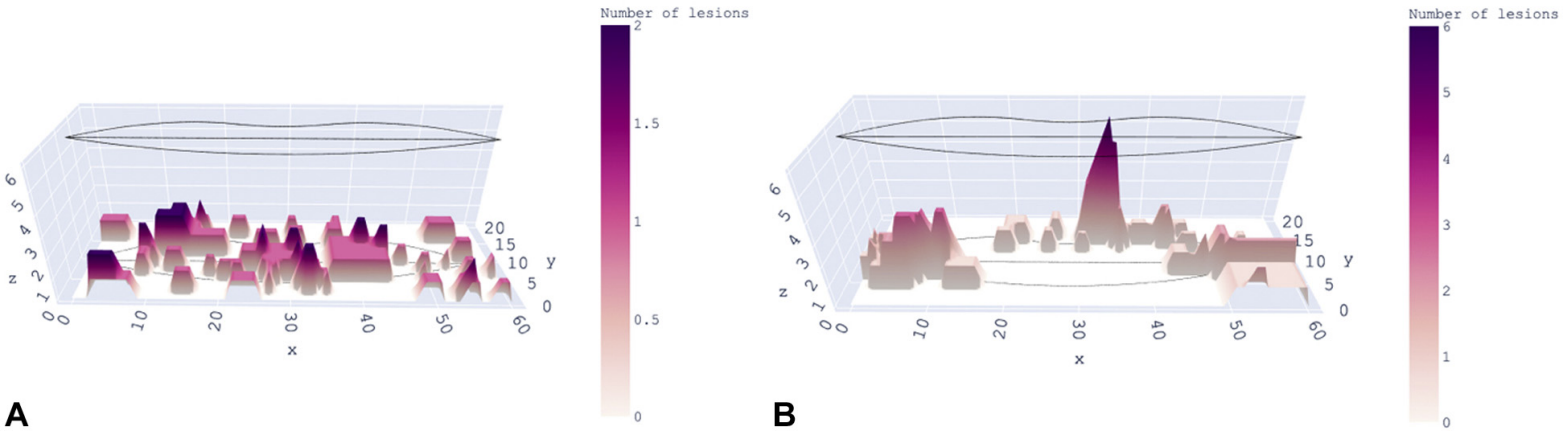
**A** and **B,** Frontal and horizontal views of outbreaks of herpes simplex virus type 1 recrudescences shown as summed surfaces. The simulation in **A** (left above) shows expected recrudescences if they were distributed randomly. This is compared with observed recrudescences in **B** (right above), where all recurrences over the years are summed together to yield a height in the vertical axis. That is, a higher peak means more events have occurred at that the site. The height of each peak represents the number of recrudescences at each site.

**Fig 3:**
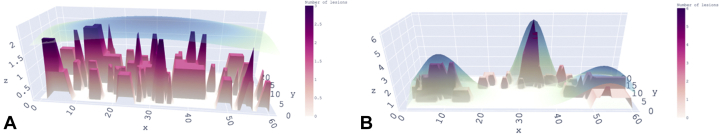
**A** and **B,** Summed herpes simplex virus type 1 outbreaks modeled by normal (Gaussian or Bell curve) functions. The fit to simulations of expected distribution of outbreaks exhibits a smooth surface over the entirety of the lips, with no peaks as shown in **A** (left above). The fit to the observed distribution of outbreaks exhibits peaks, as shown in **B** (right above). The observed distribution shows aggregations of outbreaks (**B**), which were much more concentrated in a few sites. The standard 2 dimensional normal/Gaussian function to fit the height of the surface is described.

**Fig 4:**
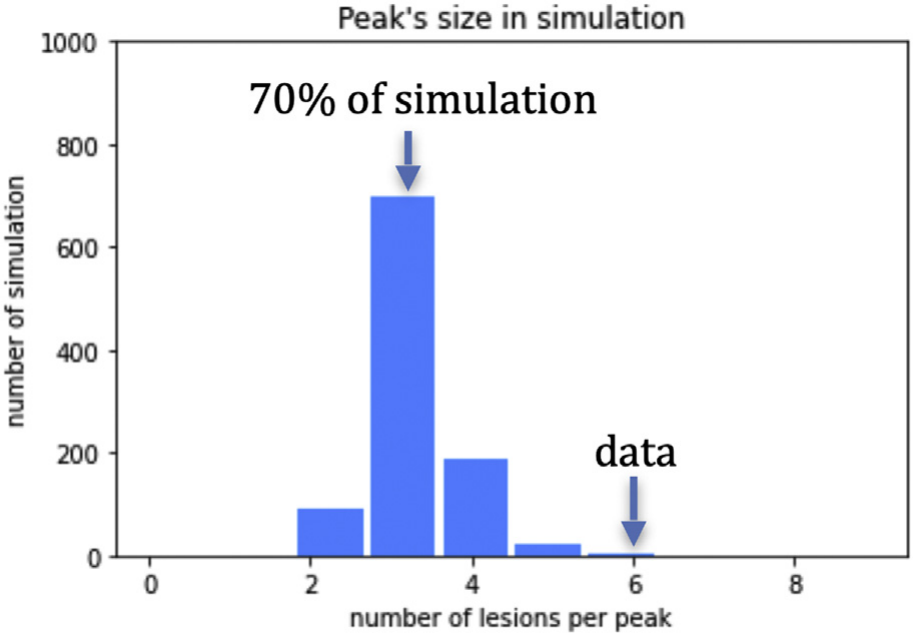
Herpes simplex virus type 1. Histogram showing expected and observed herpes simplex virus type 1 outbreaks. The number of lesions in the largest peak by simulation has a value of 3. The number of lesions forming a peak of 6 by simulation occurs 1 in a 1000 times, indicating that the observed peak with a value of 6 did not occur by chance.

There were 44 outbreaks over the 11-year span from 2008 to 2018, during which period the locations of the outbreaks, overlaps noted, were measured with millimeter precision as shown as bubbles the colors of which correspond to year of occurrence (Fig 1). Simulation runs of summed spatial distribution of outbreaks showed lesion position on a random basis. Data analysis of the observed distribution of outbreaks disclosed that observed outbreaks were not random but rather clustered at a few preferred sites. The probability that the observed distribution occurred by chance provides a *P* value of .001 (Fig 2). These findings may also be demonstrated using a Gaussian model (Fig 3). A histogram of simulated versus observed outbreaks is shown in Fig 4.

## Discussion

This uncommonly long-term case study reveals that in an immunocompetent host, the frequency of outbreaks decreased over decades. There are data that might explain the decrease. Interleukin (IL) 1α secretion from keratinocytes is higher in aged skin. The effect of higher IL-1α secretion may be augmented by lower levels of IL-1 receptor antagonist in aged skin. Stratum corneum chronic inflammatory proteins increase with age. CD8^+^ T cells increase with age and produce more tumor necrosis factor α and interferon gamma. With aging, CD4^+^ cells produce a proinflammatory T helper 17 phenotype.^2^

A survey of dental professionals disclosed that 39.6% of the respondents reported recurrent oral-labial HSV infections when they were students, whereas 23.9% of the same respondents reported recurrent infections after a gap of 12 years.^3^

The subject presented in this case had applied TCA 100% at the onset of outbreaks of HSV-1 during the first 11 years of recording frequency and labial sites. He induced superficial scarring with TCA, commonly used for aesthetic chemical peels.^4^ Having been aware of tangential shave removal of recurrent HSV, he had applied TCA 100% to block such HSV outbreaks. He was aware that creation of superficial scarring at sites of recrudescent HSV could result in dermal rather than epidermal HSV lesions^5^; however, he did not observe post-TCA subepidermal herpetic bullae. Indeed, there appeared to be no impact on frequency or site of labial outbreaks subsequent to TCA application. Comparing the frequency of outbreaks during the first 11 years with the second 11 years, the t test *P* value was .8, confirming lack of impact of TCA applications.

Upon exposure to mucosa, HSV enters sensory nerve endings, and then it moves retrograde to nerve nuclei in the ganglia. With activation, the virus moves anterograde, spreading more widely to mucosal and skin surfaces.^6^ HSV-1 DNA has been identified at healed sites of previous outbreaks, raising the possibility of ongoing immune stimulation at affected peripheral sites.^7^ In keratinocytes, HSV-1 and HSV type 2 induce IL-17c expression. Human sensory neurites grow longer and faster with more branches in the presence of IL-17c compared with its absence. IL-17c provides a neural growth and survival signal during HSV infection; still, it should be noted that most of these studies were performed with HSV type 2.^8^ However, cytolytic antiviral CD8αα cells remain at the dermoepidermal junction, lacking recirculating CCR7 and S1PR1 signals. The persistent localization of CD8αα cells at the dermoepidermal junction may serve to contain recrudescent HSV infections.^9^

It is well established that asymptomatic shedding of HSV is common in saliva in the absence of overt clinical expression.^1^ The saliva bathes the entire mouth such that clinical outbreaks of HSV might be expected to occur uniformly on and around the vermilion. They do not. Our data reveal “hot spots.” These sites of frequent recrudescence may be explained. It has been shown that some neurons allow replication of virus, while others do not.^10^ Moreover all latently infected neurons are exposed to factors known to induce reactivation; yet, only a subset undergoes recrudescence. This selective recrudescence may result from variability in the copy numbers of viral DNA among latently infected neurons.^10^

## Conflicts of interest

None disclosed.
